# CONFERD-HP: recommendations for reporting COmpeteNcy FramEwoRk Development in health professions

**DOI:** 10.1093/bjs/znac394

**Published:** 2022-11-23

**Authors:** Alan M Batt, Walter Tavares, Tanya Horsley, Jessica V Rich, Brett Williams, CONFERD-HP Collaborators, Eleanor Beck, Katarzyna Czabanowska, Gerard Fitzgerald, Elizabeth Halcomb, Karen Hauer, Deborah Hsu, Tamara Köhler, Lindy H Landzaat, Laura J Morrison, Claire Palermo, Eneline A H Pessoa, Gail Sullivan, Sean Tackett, Emma Tonkin, Riva Touger-Decker, Tim Wilkinson

**Affiliations:** Department of Paramedicine, Monash University, Melbourne, VIC, Australia; Faculty of Health, Community Studies and Public Safety, Fanshawe College, London, Ontario, Canada; Department of Paramedicine, Monash University, Melbourne, VIC, Australia; Faculty of Medicine, University of Toronto, Toronto, Ontario, Canada; The Wilson Centre, University of Toronto, Toronto, Ontario, Canada; Research Unit, Royal College of Physicians and Surgeons in Canada, Ottawa, Ontario, Canada; Faculty of Education, Queens University, Kingston, Ontario, Canada; Department of Paramedicine, Monash University, Melbourne, VIC, Australia

## Abstract

**Background:**

Competency frameworks outline the perceived knowledge, skills, attitudes, and other attributes required for professional practice. These frameworks have gained in popularity, in part for their ability to inform health professions education, assessment, professional mobility, and other activities. Previous research has highlighted inadequate reporting related to their development which may then jeopardize their defensibility and utility.

**Methods:**

This study aimed to develop a set of minimum reporting criteria for developers and authors of competency frameworks in an effort to improve transparency, clarity, interpretability and appraisal of the developmental process, and its outputs. Following guidance from the Enhancing the QUAlity and Transparency Of health Research (EQUATOR) Network, an expert panel was assembled, and a knowledge synthesis, a Delphi study, and workshops were conducted using individuals with experience developing competency frameworks, to identify and achieve consensus on the essential items for a competency framework development reporting guideline.

**Results:**

An initial checklist was developed by the 35-member expert panel and the research team. Following the steps listed above, a final reporting guideline including 20 essential items across five sections (title and abstract; framework development; development process; testing; and funding/conflicts of interest) was developed.

**Conclusion:**

The COmpeteNcy FramEwoRk Development in Health Professions (CONFERD-HP) reporting guideline permits a greater understanding of relevant terminology, core concepts, and key items to report for competency framework development in the health professions.

## Teaser

The CONFERD-HP guideline outlines a set of essential reporting criteria for developers and authors of competency frameworks in an effort to improve transparency, clarity, interpretability, and appraisal of the developmental process and its outputs. The guideline will help readers develop a greater understanding of relevant terminology, core concepts, and key items to report for competency framework development in the health professions.

fx1

## Introduction

Competency frameworks provide a description of the perceived knowledge, skills, attitudes, and other attributes required to enact professional practice competently within a given context^1^. In health professions, these are developed for several reasons, which can include defining the profession, ensuring a competent workforce, and facilitating curriculum development, systems of assessment, or professional mobility. While a significant number of competency frameworks are available in medicine, surgery, nursing, and other health professions (a previous scoping review identified 190 frameworks), and more continue to be developed, their quality may be compromised due to variability in reporting that describes clearly and sufficiently how the competency framework was developed^2^. Poor, inconsistent, or insufficient reporting practices have led to concerns regarding the validity of outputs, and threaten their utility and trustworthiness in informing downstream activities such as curriculum design and descriptions of practice^2,3^. If competency frameworks are to meet their goal, consistent and sufficient reporting is one way to improve their use and judgements of quality.

Reporting guidelines are developed to help researchers improve the completeness and transparency of their research reports^4^. A reporting guideline is defined as ‘a checklist, flow diagram, or explicit text to guide authors in reporting a specific type of research, developed using explicit methodology’^4^. Examples of reporting guidelines include the Preferred Reporting Items for Systematic reviews and Meta-Analyses (PRISMA)^5^ and the Guidance for Reporting Involvement of Patients and the Public 2 (GRIPP2)^6^. As part of a broader programme of research on competency framework development, the need for improved reporting guidance for those developing competency frameworks was identified^2^. This guidance—in the form of a reporting guideline—could help to improve the utility and validity of reported competency frameworks^7^. It will also help those who wish to use competency frameworks (as a type of quality appraisal), as well as those undertaking peer review and editorial review of manuscripts describing the development of competency frameworks. Evidence demonstrates the use of reporting guidelines increases methodological transparency and uptake of research findings^7^, which is a key component in the translation of research findings into clinical practice, and education.

The aim of this study was to develop an evidence- and consensus-based reporting guideline to aid the evaluation of health professions-focused competency frameworks.

## Methods

This study followed the steps outlined by the ‘Enhancing the QUAlity and Transparency Of health Research’ (EQUATOR) network for developing reporting guidelines in health research^4^. This framework has been used successfully to develop reporting guidelines for other areas of health research^5,8^. The reporting guideline was developed in four stages, including: stage 1, project launch (research team established, expert advisor recruited, protocol drafted, and ethics approval); stage 2, knowledge synthesis (comprehensive literature and guideline review to inform and identify potential reporting items); stage 3, consensus process (panel of developers, journal editors, regulators, and end users recruited to complete a Delphi study and virtual workshops); stage 4, developing the reporting guideline (initial reporting guideline drafted to solicit feedback from the expert panel, and feedback used to create the final reporting guideline).

**Fig. 1:**
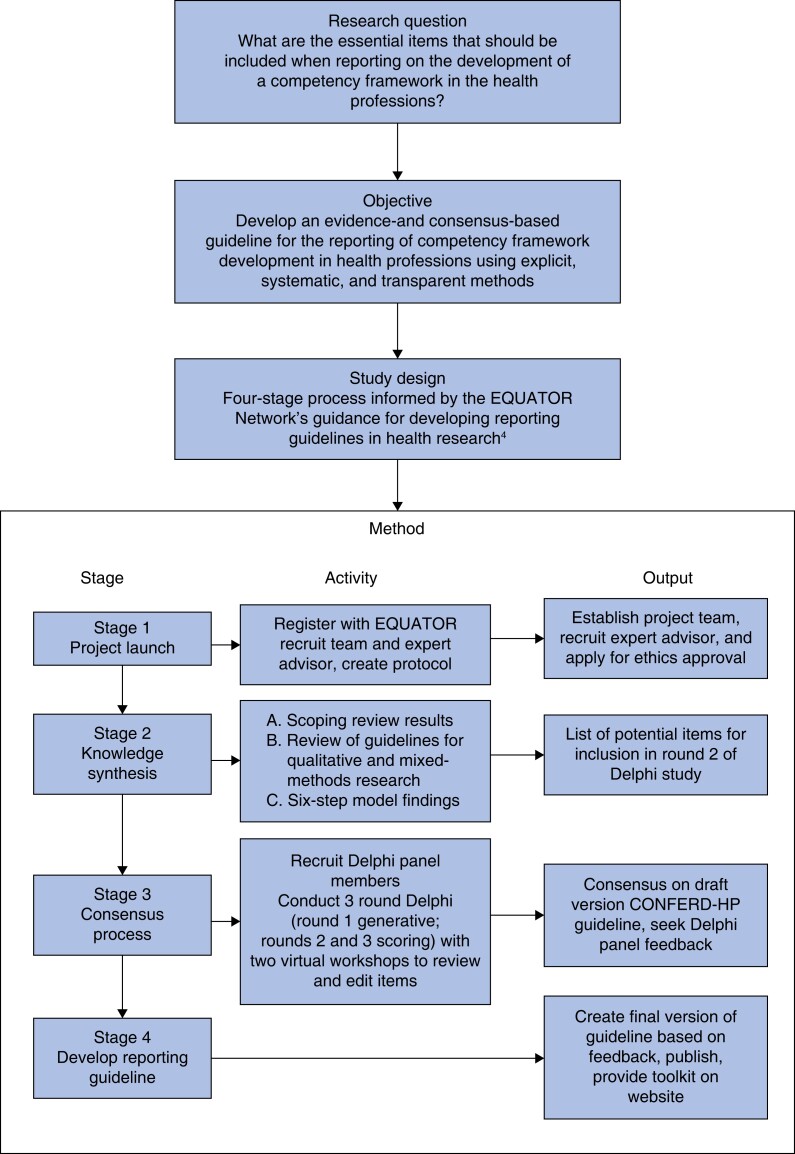
Stages of the CONFERD-HP study

See *Fig. 1* for a schematic of these stages. Details on the activities at each stage are described below.

## Stage 1: project launch

### Establish team, expert advisor, and protocol

A core project team (A.M.B., W.T., J.V.R., and B.W.) was established and were responsible for drafting the protocol and the day-to-day operations of the project. The team members provided expertise in competency framework development and evaluation, and in health profession competency-based education. The team engaged a methodological advisor (T.H.) to guide both the consensus methodology and development of the reporting guideline. This advisor guided the team in the conduct of this study, including the literature review; the nomination of participants for the Delphi study; reviewing the checklist items for inclusion in the Delphi study, providing feedback after each round of the survey (e.g. interpreted results of the previous round and approved content for the next round); and contributed to the production and reporting of the final reporting guideline. The development of the COmpeteNcy FramEwoRk Development in Health Professions (CONFERD-HP) reporting guideline was registered on the EQUATOR Network website in December 2019^9^.

## Ethics approval

The Monash University Human Research Ethics Committee provided ethics approval for this study in June 2021 (#27484).

## Stage 2: knowledge synthesis

Results from a scoping review of competency frameworks were used to inform this study^2^. The scoping review aimed to understand how health professions developed competency frameworks and then to consider these activities against existing development guidance^2^. The major aspects and characteristics of the competency framework development process reported across 190 studies were reviewed and extracted using a standardized data-collection form. This included items potentially relevant for inclusion in a reporting guideline (e.g. rationale for development, rationale for selection of methods, and declaration of funding source)^2^. All published checklists and guidelines on the EQUATOR Network website were then reviewed (421 at the time of search (January 2020)), to identify reporting guidelines related to qualitative and mixed-methods data collection. Several guidelines were extracted in full and entered into a spreadsheet. Finally, elements of a contemporary six-step competency framework development model that did not feature in the existing literature were leveraged. This six-step model furthers existing development guidance through the use of theoretical or conceptual approaches, as well as the use of mixed or multiple methods of data collection^2^. The three sources of extracted data were combined into a single spreadsheet and a key list was generated that was cross-referenced with each source.

## Stage 3: consensus strategy

### Delphi panel

The consensus approach was guided by traditional Delphi methodology. A purposive sampling strategy was employed, aimed at recruiting panel members that represented users of the guideline^10,11^. This meant attending to diversity of representation across anticipated intended and end-users, professions, contexts, and stakeholders^10,12^. To begin, intended users were defined as those who will *use the reporting guideline* (e.g. authors, those developing competency frameworks, journal editors, and regulators) and end users as those who will *use the reported competency framework* (e.g. health professionals and educators). Broad representation was sought by targeting authors of competency frameworks, editors of journals that publish competency frameworks, and health profession regulators (described in further detail below). Finally, a virtual approach was employed to allow experts who were dispersed globally to participate^13,14^, and to accommodate restrictions imposed by the ongoing COVID-19 pandemic (the guideline was developed throughout 2021). Recruitment of 30 panel members was anticipated, with detailed sampling strategy outlined below.

#### Intended users: authors

All authors of reports of competency framework development identified in the scoping review^2^ that had a corresponding email address were invited to participate. Additional authors who were not represented in the scoping review but had authored a report of competency framework development since the publication of the review were also invited. These authors were identified through database searches for papers related to competency framework development in health professions. Authors from all countries, disciplines, and organizations identified were invited with no exclusion criteria.

#### Intended users: journal editors

Journals in which competency frameworks were published were identified and ranked according to the frequency of publication of frameworks. Editors from the top-five most productive journals (having published five or more competency frameworks) were invited to participate.

#### Intended users: health professions regulators

Representatives of a number of national regulatory bodies across health professions who developed or provided oversight for the development of competency frameworks were invited. These were identified through the scoping review.

#### End users

Representatives of professional associations, individual health professionals (including physicians, surgeons, nurses, pharmacists, dietitians, therapists, and others), and educators/faculty members, identified through the research team and the results of the scoping review, were invited (e.g. associations that endorsed guidelines).

All participants were recruited via email invitation in July 2021 which outlined the objective of the study, study design, participation details, and level of commitment. Consent forms were also included in the invitation.

### Survey development

The Delphi study included three rounds: an initial generative, open-ended survey-based round, and two subsequent rounds of item scoring online surveys conducted using the edelphi.org website (with Google Form and Excel versions available in case of technical issues). Research team members tested but did not contribute data. Prior to deployment, surveys were piloted for content and clarity with the core project team and expert advisor. Participants were sent an initial invite to each round, a reminder at 1 week, and a final reminder with 3 days remaining to optimize participation. Each survey round was open for 3 weeks with 2 weeks between each round for analysis and synthesis.

### Round 1 of Delphi

Delphi panel members were asked one non-identifying demographic question related to their role in competency framework development. This was asked to gain insight into the composition of the panel in relation to their roles, and to ensure diversity in responses. Panel members where then asked to indicate (via free-text responses) the essential items that should be included when reporting the development of a competency framework. Specifically, they were asked: ‘As a consumer, when reading a report/publication about the development of a competency-related framework, at a minimum, what elements would require reporting in order to imbue trust in the competency framework itself?’ In keeping with traditional approaches and as a means of reducing bias, this open-ended approach was implemented rather than providing a list of items that were informed by the knowledge synthesis (described above).

### Round 1 analysis

Results from Round 1 were analysed using continuous content analysis, whereby the data were analysed to inductively generate lists and categories of minimum required reporting items^15^. For example, items suggested by panel members related to target audience, title, and definitions were categorized under ‘Background information’. One member of the research team (A.M.B.) then collapsed redundant or repetitive concepts or items within categories into a single reporting item (see *Table S1* ). The potential reporting items generated during the knowledge synthesis were then added to the list presented to the panel in round 2 of the Delphi process. The revised list of reporting items and brief explanations was distributed among the research team and expert advisor for review and feedback prior to distribution to Delphi panel members for round 2.

### Round 2 of Delphi

Using a 7-point Likert scale (1 = strongly disagree (exclude item), 7 = strongly agree (include item)), Delphi panel members were asked to indicate their agreement with inclusion of each proposed reporting item in a reporting guideline. Each proposed reporting item included detail on whether it originated from the knowledge synthesis exercise (stage 2) or round 1 of the Delphi, and, if it was from round 1, how many panel members suggested the item. An optional text-box response option was also included where respondents could provide feedback and suggestions to improve the clarity of wording for each item. This feedback was visible to other members of the panel in an anonymous format. The feedback was used to clarify wording for round 3, and to identify items considered duplicate by panel members. This feedback was distributed to all panel members in advance of round 3, the virtual workshops, and final feedback.

### Round 3 of Delphi

Delphi panel members completed a third round of scoring of reworded and clarified discrepant items based on round 2 feedback. Delphi panel members were asked to rate their agreement with each of the reworded items using the same 7-point Likert scale as above. Each survey item included an optional text box where respondents could provide further comments.

### Round 2 and round 3 analysis

The approach to round 2 and 3 survey analysis involved calculating the average scores for the group to feed back to individual participants in the following Delphi round. Seventy per cent agreement was established a priori for each of the reporting items as a threshold for consensus among the Delphi panel members. This rule required that at least 70 per cent of the respondents indicated that they either ‘agreed’ or ‘strongly agreed’ (values of 6 or 7 on the Likert scale) with the inclusion of the item as an essential requirement within the reporting guideline. If agreement was less than 70 per cent the item was considered discrepant and brought forward for discussion at the workshops. If agreement was just over 70 per cent or the distribution of responses was not clearly in favour of agreement (e.g. not mainly values of 6 or 7 on the Likert scale), the item was brought forward for discussion at the virtual workshops. In addition, the free-text comments provided by Delphi panel members were analysed for content and used to inform decisions for either the merging or rewording of draft items generated at the end of each round. The Delphi panel members were provided with a detailed report at the end of each round, which contained the level of agreement for each item and a summary of the free-text comments.

### Workshops

The aim of this stage in reporting guideline development was to discuss and gain consensus on the items for inclusion in the final reporting guideline^4^. To this end, two 2-hour online virtual workshops were held in November 2021, facilitated by several members of the research team (A.M.B., J.V.R., and W.T.) and the expert advisor (T.H.). All participants who participated in at least one of the steps above were invited. Attendees reviewed the items resulting from the Delphi rounds, and provided feedback on the wording to promote clarity, and sequence of each item. In the workshops, participants and the research team worked in a live synchronous environment whereby edits were made as suggested by participants. Disagreement was resolved by discussion. Workshops were held via Zoom to facilitate attendance across multiple time zones and to comply with COVID-19 restrictions. Workshops were recorded and detailed notes were taken by the research team.

### Draft guideline

A draft reporting guideline was developed based on the results of the knowledge synthesis, consensus process, feedback from workshop participants, and notes taken by the research team. The draft was circulated to all Delphi panel members to receive further feedback on item wording, clarity, sequence of items, and categories within the checklist. The research team then used the feedback provided by Delphi panel members on the draft reporting guideline to inform the final version of the guideline.

### Measures to promote trustworthiness

To promote trustworthiness the research team strived to demonstrate dependability, credibility, confirmability, and transferability^16^. Diversity was sought in representation across users, professions, contexts, and stakeholders, to improve dependability^10^. Credibility in the process was ensured by providing ongoing feedback from Delphi panel members to each other as a form of member checking^10,17^. The Delphi study was reported in detail in line with the Guidance on Conducting and REporting DElphi Studies (CREDES) reporting guideline^18^, to improve confirmability.

### Participant stipends and payments

Participants were not remunerated for their participation in this study.

## Results

### Stage 2: knowledge synthesis

Analysis of the scoping review findings related to reporting quality revealed several inconsistencies in the reporting of competency framework development (*Table 1*)^2^. The search of the EQUATOR Network website for mixed-methods and qualitative reporting guidelines resulted in the identification of six relevant guidelines^18–23^. A total of 157 individual items were abstracted from these guidelines. Finally, the competency framework development guidance was reviewed which resulted in the identification of 31 potential reporting items^1,2^. When cross-referenced and collapsed, this resulted in a synthesized list of 17 potential reporting items for mapping against the panel responses from round 1 of the Delphi study.

**Table 1:** Reporting of items in scoping review

Potential reporting item described	Competency frameworks reporting the item
Geographical location identified	190 (100)
Intended profession	190 (100)
Rationale for developing framework	190 (100)
Funding source(s) declared	110 (58)
Timeframe for development of framework	81 (43)
Rationale for choice of method(s) used	79 (42)
Title contains term ‘competency’	70 (37)
Evaluation planned or recommended	66 (35)
Title contains term ‘competencies’	65 (34)
Title contains term ‘competency framework’	30 (16)
Partial rationale for choice of methods	27 (14)
Patient engagement in development process	21 (11)
Triangulation of data	18 (9)
Title contains term ‘competency model’	9 (5)
Title contains term ‘competency standard’	9 (5)
Evaluation conducted	7 (4)

Values are *n* (%).

### Stage 3: consensus process

**Fig. 2:**
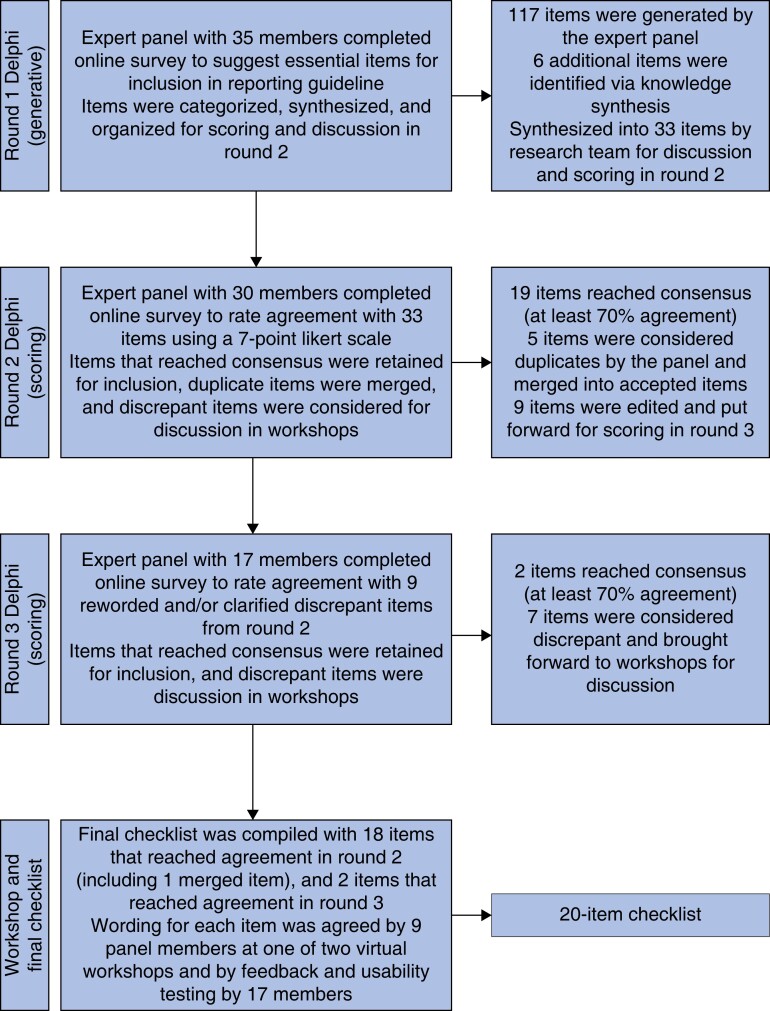
Results of the Delphi study and virtual workshops

A total of 155 individuals were invited to participate on the panel. Some 35 individuals completed round 1 (generative phase) of the Delphi study (23 per cent overall response rate) (*Table S1*). Subsequently, 30 of 35 completed round 2 (86 per cent response rate), and 17 of 35 completed round 3 (49 per cent response rate). Nine of 35 panel members attended the workshops (26 per cent response rate), and final feedback was received from 17 panel members (49 per cent response rate). Several participants identified themselves as having multiple roles related to competency framework development (*Table 2*, *Fig. 2*).

**Table 2:** Characteristics of the expert panel in the study

Role in competency frameworks*	Number of participants fulfilling role (% of panel) in Delphi round 1	Number of participants fulfilling role (% of panel) in virtual workshops	Number of participants fulfilling role (% of panel) in final feedback
Developer	25 (72)	9 (26)	12 (34)
Journal Editor	9 (26)	1 (3)	3 (9)
Regulator of a healthcare profession	4 (12)	1 (3)	2 (6)
End user (e.g. educator, healthcare professional)	19 (55)	8 (23)	15 (43)
**Participant profession/area of focus**			
ȃMedicine (incl. surgery)	15 (43)	4 (11)	7 (20)
ȃNursing	7 (20)	1 (3)	2 (6)
ȃMultidisciplinary	7 (20)	1 (3)	3 (9)
ȃAllied health professions (pharmacy, dietetics, paramedic, occupational therapy)	6 (18)	3 (9)	5 (15)
**Geographical location of participants**			
ȃNorth America	15 (43)	6 (18)	7 (20)
ȃAustralasia	8 (23)	1 (3)	5 (14)
ȃEurope	9 (26)	2 (6)	3 (9)
ȃAsia	2 (6)	0	0
ȃSouth America	1 (3)	0	1 (3)

Rounds 2 and 3 were anonymous and no demographic information was gathered. *Some panel members held more than one role and therefore the total is greater than the number of participants. Values are n (%) unless otherwise indicated.

### Round 1

An initial list of 117 suggested reporting items was generated. Following categorization and synthesis, a list of 27 reporting items was identified. This list was mapped against the knowledge synthesis findings (stage 2), which resulted in an additional six unique items for a total of 33 potential reporting items.

### Round 2

A panel of 30 expert participants scored 33 potential reporting items from round 1. Five participants did not complete the survey. Overall, 19 items reached 70 per cent agreement, five items were considered duplicate and were merged with agreed items (e.g. the panel suggested merging four items on the reporting of different methods to one tiered item addressing ‘methods’—item 9c). Nine items were considered discrepant and brought forward for consideration in round 3. Edits were suggested to the wording of all but one item (item 1a). (*Table S2*).

### Round 3

A panel of 17 expert participants scored nine discrepant reporting items from round 2. Overall, two items (both rephrased based on round 2 feedback) reached 70 per cent agreement (items 2 and 7). Seven items remained discrepant and were brought forward for discussion in the workshops. (*Table S2*).

### Workshops and initial draft

A total of nine participants attended one of two virtual workshops. Participants were exclusively female, largely held senior academic ranks (e.g. Associate Professor, Professor, and Chair), and represented multiple health professions (e.g. medicine, nursing, dietetics, and pharmacy). In these workshops, which were facilitated by the expert advisor and members of the research team, an initial draft guideline was presented, and revisions were suggested to the wording of items. Revisions were suggested to the wording of all but four of the items between both workshops (items 1a, 3, 11, and 14). Two items were merged based on workshop feedback.

### Revised draft guideline and feedback

A revised draft guideline was generated that contained 20 reporting items, and this was circulated to the Delphi panel for feedback. Feedback was received from 17 panel members across four roles and eight disciplines, and 16 further elected to be identified as collaborators on the guideline. A sample of four participants, who volunteered, ‘tested’ the reporting guideline for usability by applying it with development, peer-review, and quality-appraisal contexts in mind. All items were reviewed and edited for clarity and sequence based on the feedback from the Delphi panel. A copy editor edited the final checklist to ensure clarity, consistency, and grammatical accuracy.

## Discussion

The CONFERD-HP reporting guideline was developed using an evidence-informed approach as outlined by the EQUATOR Network^4^. The CONFERD-HP reporting guideline is intended to guide the essential items that should be reported when describing the development of competency frameworks in the health professions, including medical specialities, surgical specialties, nursing, allied health, and others. The final reporting guideline checklist is presented in *Table 3* and includes 20 items. It consists of 18 items that reached agreement in round 2, along with two additional items that were modified for inclusion after round 3. The checklist items encompass the following categories: basic information (e.g. title); background information (e.g. purpose); development process (e.g. methods); testing; funding; and conflicts of interest. An explanation and elaboration statement is being prepared, which will provide readers with a comprehensive explanation and rationale, as well as examples of good reporting, for each item in the reporting guideline.

**Table 3:** CONFERD-HP checklist

Section/topic	Item	Checklist item
**Title and abstract**		
ȃTitle	1a	Identification as a competency framework in the title
	1b	Identification of intended profession and level of practice/stage of training in title
ȃStructured abstract/summary	2	Structured summary that includes intended user(s) and use(s) of the framework, the purpose of the framework, the development process and methods used
ȃDefinition(s)	3	Defined or referenced definitions for competence, competency, and other key terms used to promote understanding of the framework
**Framework development**		
ȃRationale and justification	4	Description of rationale and justification for the development of the framework, including supportive references where possible
ȃPurpose and use	5a	Description of the purpose of the framework
	5b	Description of the intended use(s) of the framework
	5c	Description of the intended user(s) of the framework
ȃDeveloper group	6	Description of the qualifications and expertise of those leading the development of the framework
ȃOversight/governance group	7	Description of the group that had oversight of the framework, the purpose and expertise of the group members, how they reviewed the work, and/or contributed to the development
ȃTheoretical/conceptual approach(es)	8	Description of theoretical/conceptual approach(es) used to develop the framework, including references and rationale for their use
**Development process**		
ȃProcess and methods	9a	Description of each step of the development process
	9b	Description of how existing literature was gathered and used to inform the competency framework development. Provide a list of references used
	9c	Description of all methods used throughout the development process, including associated reference(s) and details of any modifications
ȃEnd-user contributions	10	Description of all stakeholders, including end users of both the framework (e.g. the professional group consulted) and the services (e.g. patients/consumers and other healthcare professionals) who contributed to the development process, how they were selected (with considerations of equity, diversity, and inclusion), and how they participated.
ȃEthics	11	Description of ethical considerations and approvals obtained where applicable
**Evaluation and implementation**		
ȃEvaluation	12	Description of the approach for evaluating the draft competency framework, including how feedback from stakeholders was gathered and used
ȃImplementation	13	Suggestion for how the framework should be implemented and in what settings
**Funding and conflicts of interest (COI)**		
ȃFunding	14	Description of all funding sources and other support received for the development of the framework and the role of the funder(s)
ȃCOI	15	Description of how COI were considered and managed in the development process

The reporting guideline can assist those developing competency frameworks in the development process, support journal editors and peer reviewers when considering frameworks for publication, and help end users (e.g. educators, professionals, and regulators) understand the scope, rigour, and trustworthiness of a competency framework, and inform decisions around its utility and applicability for their intended purposes. When the reporting guideline is applied, it results in a clear, explicit description of processes and procedures that were used when developing a competency framework, and access to the resources and evidence used to formulate each recommended item. The CONFERD-HP reporting guideline is not intended to be a prescriptive or linear format for reporting competency framework development. Rather, each item should be presented, and sufficient elaboration provided somewhere in the reporting of the competency framework development process.

The CONFERD-HP study should be interpreted in light of some limitations. Firstly, the Delphi panel response rate represented 23 per cent of those invited. While there is no sample size calculation for the Delphi method, it is generally accepted that a larger sample may increase the reliability of the group’s judgements, and more than 12 participants are recommended^24^—a threshold which was met in all three rounds. More importantly, panel members represented a diverse sample of all potential end users of the reporting guideline, With input from experts in medicine, surgery, nursing, allied health professions, and multidisciplinary fields such as public health, the study managed to gained input from the full range of the population the guidance is intended to influence^24^. Secondly, not all Delphi panel members were able to participate in all rounds of the Delphi. Unfortunately, schedule and COVID-19 demands placed on Delphi panel members made this a challenge. In an effort to reduce the effects of attrition, all members of the Delphi panel were engaged at several points—Delphi rounds, workshops, and an opportunity to provide final feedback via email. Thirdly, important items may have been missed in the initial literature review (stage 2), but every effort was made to minimize this possibility by examining relevant reporting guidelines and engaging the expertise of the Delphi panel in round 1. Thirdly, some items identified from the knowledge synthesis were not considered relevant by the Delphi panel. In addition, the panel suggested 10 items more than the knowledge synthesis had generated. This may be partly due to wording (as evidenced by reworded items in round 3 gaining agreement) but is most likely because the perspectives of the panel are informed by their real-world experiences developing, reporting on, and publishing competency frameworks. The expert insight provided by the Delphi panel ensures the reporting guideline will be useable and useful. Finally, like any other reporting guideline, the CONFERD-HP reporting guideline is an evolving document that will require ongoing evaluation, improvement, and updating. There may also need to be adaptations across professions as the framework is used. For example, some professions (or specialties within professions) may require consideration of items not included in this guideline. The statement will be revised in the future based on user feedback, results of evaluations, and improved guidance on reporting guidelines. Those who use the CONFERD-HP reporting guideline are encouraged to submit comments via the CONFERD-HP website (www.conferd-guideline.org).

Use of the CONFERD-HP reporting guideline will be encouraged by journals by contacting the editors of journals that have published competency frameworks to elicit their support and inform national and international competency framework developers about the reporting guideline.

## Supplementary Material

znac394_Supplementary_Data

## Data Availability

The knowledge synthesis results, Delphi scoring results, and draft feedback reports are available from Alan Batt (alan.batt1@monash.edu)

## Collaborators

Eleanor Beck (Faculty of Science, Medicine and Health, University of Wollongong, Australia); Katarzyna Czabanowska (International Health Department, Maastricht University, Maastricht, the Netherlands); Gerard Fitzgerald (School of Public Health and Social Work, Queensland University of Technology, Australia); Elizabeth Halcomb (School of Nursing, University of Wollongong, Australia); Karen Hauer (Competency Assessment and Professional Standards, University of California at San Francisco, San Francisco, California, USA); Deborah Hsu (Division and Service Chief, Pediatric Emergency Medicine, Baylor College of Medicine and Texas Children’s Hospital, Texas, USA); Tamara Köhler (Division of Pharmacoepidemiology and Clinical Pharmacology, Utrecht Institute for Pharmaceutical Sciences; Department of Pharmaceutical Sciences, Faculty of Science, Utrecht University, the Netherlands); Lindy H. Landzaat (University of Kansas Medical Center, Kansas, USA); Laura J. Morrison (Yale School of Medicine, New Haven, Connecticut, USA); Claire Palermo (Faculty of Medicine, Nursing and Health Sciences; Monash Centre for Scholarship in Health Education, Monash University, Australia); Eneline A. H. Pessoa (Faculdade Pernambucana de Saúde Recife, Brazil); Gail Sullivan (UConn Center on Aging, University of Connecticut School of Medicine, USA); Sean Tackett (Johns Hopkins Bayview Medical Center, Baltimore, MD, USA); Emma Tonkin (University of South Wales, Pontypridd, UK); Riva Touger-Decker (School of Health Professions and Division of Nutrition, Rutgers School of Dental Medicine, Rutgers University, Newark, New Jersey, USA); Tim Wilkinson (University of Otago, Christchurch, New Zealand).

## References

[1] Batt A , Williams B, Rich J, Tavares W. A six-step model for developing competency frameworks in the healthcare professions. Front Med 2021;8:2601 10.3389/fmed.2021.789828 PMC8713730 34970566

[2] Batt AM , Tavares W, Williams B. The development of competency frameworks in healthcare professions: a scoping review. Adv Health Sci Educ 2020;25:913–987 10.1007/s10459-019-09946-w 31797195

[3] Batt AM , Williams B, Brydges M, Leyenaar M, Tavares W. New ways of seeing: supplementing existing competency framework development guidelines with systems thinking. Adv Health Sci Educ Theory Pract 2021;26:1355–1371 34003391 10.1007/s10459-021-10054-x

[4] Moher D , Schulz KF, Simera I, Altman DG. Guidance for developers of health research reporting guidelines. PLoS Med 2010;7:e1000217 20169112 10.1371/journal.pmed.1000217 PMC2821895

[5] Page MJ , Moher D, Bossuyt PM, Boutron I, Hoffmann TC, Mulrow CD et al PRISMA 2020 Explanation and elaboration: updated guidance and exemplars for reporting systematic reviews. BMJ 2021;372:n160 33781993 10.1136/bmj.n160 PMC8005925

[6] Staniszewska S , Brett J, Simera I, Seers K, Mockford C, Goodlad S et al GRIPP2 Reporting checklists: tools to improve reporting of patient and public involvement in research. BMJ 2017;358:j3453 28768629 10.1136/bmj.j3453 PMC5539518

[7] Simera I , Moher D, Hirst A, Hoey J, Schulz KF, Altman DG. Transparent and accurate reporting increases reliability, utility, and impact of your research: reporting guidelines and the EQUATOR network. BMC Med 2010;8:24. 20420659 10.1186/1741-7015-8-24 PMC2874506

[8] Tricco AC , Lillie E, Zarin W, O’Brien KK, Colquhoun H, Levac D et al PRISMA Extension for Scoping Reviews (PRISMA-ScR): checklist and explanation. Ann Intern Med 2018;169(7):467–473. doi: 10.7326/M18-0850 30178033 10.7326/M18-0850

[9] EQUATOR Network . Reporting guidelines under development for other study designs: CONFERD-HP. https://www.equator-network.org/library/reporting-guidelines-under-development/reporting-guidelines-under-development-for-other-study-designs/#CONFERD (accessed 12 February 2020)

[10] Cornick P . Nitric oxide education survey—use of a Delphi survey to produce guidelines for training neonatal nurses to work with inhaled nitric oxide. J Neonatal Nurs 2006;12:62–68

[11] Trevelyan EG , Robinson N. Delphi methodology in health research: how to do it? Eur J Integr Med 2015;7:423–428

[12] Sinha IP , Smyth RL, Williamson PR. Using the Delphi technique to determine which outcomes to measure in clinical trials: recommendations for the future based on a systematic review of existing studies. PLoS Med 2011;8:e1000393 21283604 10.1371/journal.pmed.1000393 PMC3026691

[13] Msibi PN , Mogale R, De Waal M, Ngcobo N. Using e-Delphi to formulate and appraise the guidelines for women’s health concerns at a coal mine: a case study. Curationis 2018;41:1–6 10.4102/curationis.v41i1.1934 PMC6191662 30326704

[14] Hall DA , Smith H, Heffernan E, Fackrell K, Core Outcome Measures in Tinnitus International Delphi (COMiT’ID) Research Steering Group. Recruiting and retaining participants in e-Delphi surveys for core outcome set development: evaluating the COMiT’ID study. PLoS One 2018;13:e0201378 30059560 10.1371/journal.pone.0201378 PMC6066228

[15] Krippendorff K . Content analysis: an introduction to its methodology. London: Sage, 2004.

[16] Polit D , Beck C, Hungler B. Essentials of nursing research: methods, appraisal, and utilization. 5th ed. Philadelphia: Lippincott Williams and Wilkins, 2001.

[17] Engels TCE , Powell Kennedy H. Enhancing a Delphi study on family-focused prevention. Technol Forecast Soc Change 2007;74:433–451.

[18] Jünger S , Payne SA, Brine J, Radbruch L, Brearley SG. Guidance on Conducting and REporting DElphi Studies (CREDES) in palliative care: recommendations based on a methodological systematic review. Palliat Med 2017;31:684–706 28190381 10.1177/0269216317690685

[19] Tong A , Sainsbury P, Craig J. Consolidated criteria for reporting qualitative research (COREQ): a 32-item checklist for interviews and focus groups. Int J Qual Health Care 2007;19:349–357 17872937 10.1093/intqhc/mzm042

[20] Staniszewska S , Brett J, Mockford C, Barber R. The GRIPP checklist: strengthening the quality of patient and public involvement reporting in research. Int J Technol Assess Health Care 2011;27:391–399 22004782 10.1017/S0266462311000481

[21] O’Brien BC , Harris IB, Beckman TJ, Reed DA, Cook DA. Standards for reporting qualitative research: a synthesis of recommendations. Acad Med 2014;89:1245–1251 24979285 10.1097/ACM.0000000000000388

[22] Leech NL , Onwuegbuzie AJ. Guidelines for conducting and reporting mixed research in the field of counseling and beyond. J Couns Dev 2010;88:61–69

[23] O’Cathain A , Murphy E, Nicholl J. The quality of mixed methods studies in health services research. J Health Serv Res Policy 2008;13:92–98 10.1258/jhsrp.2007.007074 18416914

[24] Murphy MK , Sanderson C, Black NA, Askham J, Lamping DL, Marteau T et al Consensus development methods, and their use in clinical guideline development. Health Technol Assess 1998;2:i–iv, 1–88 9561895

