# Supplementary material for: CONFERD-HP: recommendations for reporting COmpeteNcy FramEwoRk Development in health professions
Source: Br J Surg. 2022 Nov 23;110(2):233–41. doi: 10.1093/bjs/znac394 (PMC10364529; doi:10.1093/bjs/znac394)
Supplement: znac394_Supplementary_Data [file znac394_supplementary_data.docx]

**CONFERD-HP: Recommendations for reporting COmpeteNcy FramEwoRk Development in Health Professions.**

Alan M. Batt^1,2^, Walter Tavares^1,3,4^, Tanya Horsley^5^, Jessica V. Rich^6^, Brett Williams^1^ and the CONFERD-HP Collaborators

^1^Department of Paramedicine, Monash University, Australia.

^2^Fanshawe College, Ontario, Canada.

^3^Faculty of Medicine, University of Toronto, Ontario, Canada

^4^The Wilson Centre, University of Toronto, Ontario, Canada

^5^Royal College of Physicians and Surgeons in Canada, Ottawa, Ontario, Canada

^6^Queens University, Ontario, Canada

**Corresponding author.** Alan M. Batt, Dept. of Paramedicine, Building H, McMahons Road, Frankston, Vic, 3199, Australia; Email: [alan.batt1@monash.edu](mailto:alan.batt1@monash.edu) **ORCID ID** 0000-0001-6473-5397**; Twitter** @alan_batt

**Supplementary Materials - Index**

| **Supplementary Figures and Tables** |  |
| --- | --- |
| Table S1. Coding of items from Round 1 Delphi | *pag. 2* |
| Table S2. Scoring of items in Delphi Rounds 2 and 3 | *pag. 3* |

**Table S1 – Coding of items from Round 1 Delphi**

| **Category assigned** | **Item suggested by panel in Round 1** | **Code assigned** | **Combined item for Round 2 from collapsed codes** |
| --- | --- | --- | --- |
| Background | What stakeholders were involved in its development | Stakeholder involvement | Clearly outline stakeholders involved, how they were selected, how they were involved |
| Background | Involvement of multiple different perspective/stakeholder | Stakeholder involvement |  |
| Background | A list of stakeholders involved in the development of the framework and acknowledgement of their contribution | Stakeholder involvement |  |
| Background | People involved in creating the framework | Stakeholder involvement |  |
| Background | State groups of stakeholders and how they were incorporated | Stakeholder involvement |  |
| Background | Identify key stakeholders of the competency framework. | Stakeholder involvement |  |

**Table S2 – Scoring of items in Delphi Rounds 2 and 3**

| **Item #** | **Item text** | **% of agreement in Round 2** | **% of agreement in Round 3** | **Final checklist item informed** |
| --- | --- | --- | --- | --- |
| 1 | Define competency and other terms used in order to promote understanding of the framework/profile | 93.3 | N/A | 3 |
| 2 | State target audience/profession in title | 73.4 | N/A | 1b |
| 3 | Identify the report as a competency framework OR competency profile in the title | 76.7 | N/A | 1a |
| 4 | Provide a structured summary/abstract | 56.7 – reworded and sent to Round 3 | 87.5 | 2 |
| 5 | State the background and stance of the developers, expertise of the developers | 73.4 | N/A | 6 |
| 6 | Describe the diverse steering group who led the project | 63.3 – reworded and sent to Round 3 | 87.5 | 7 |
| 7 | Describe the need/rationale for the development of the framework/profile | 73.3 | N/A | 4 |
| 8 | Describe the boundaries of the development process set by practicalities (e.g. timeframes, budget) | 33.3 – reworded and sent to Round 3 | 6.3 | N/A |
| 9 | Outline the intended uses of the framework/profile | 100 | N/A | 5b |
| 10 | Describe the target audience for the framework/profile | 83.3 | N/A | 5c |
| 11 | Describe how to use or implement the framework/profile | 86.6 | N/A | 13 |
| 12 | State the contexts of use of the framework/profile | 73.3 | N/A | 5b |
| 13 | Clearly outline stakeholders involved, how they were selected, how they were involved | 76.6 | N/A | 10 |
| 14 | Describe the purpose of the framework/profile | 73.3 | N/A | 5a |
| 15 | Describe the theoretical or conceptual approaches used in the development process | 70 | N/A | 8 |
| 16 | Outline the development process in detail | 86.7 | N/A | 9a |
| 17 | Describe a clear link to an analysis of practice | 20 | 7.1 | N/A |
| 18 | If qualitative methods were used, describe each in appropriate detail | 76.7 | 76.7 – merged item | 9c |
| 19 | If quantitative methods were used, describe each in appropriate detail | 66.7 – suggested merge with item 18 | 76.7 – merged item | 9c |
| 20 | If consensus methods were used, describe in appropriate detail, outline who participated, how they were selected | 63.3 – suggested merge with item 18 | 76.7 – merged item | 9c |
| 21 | Conduct a literature review and describe in appropriate detail | 73.4 | N/A | 9b |
| 22 | Describe multidisciplinary and patient input if applicable | 73.4 | N/A | 10 |
| 23 | Outline ethics approval(s) for methods that involve human participants | 70 | N/A | 11 |
| 24 | Provide a rationale for each choice of method(s) | 80 - suggested merge to item 18 | 76.7 – merged item | 9c |
| 25 | If mixed methods were used, provide appropriate detail on design, sequence, integration etc. | 73.3 – suggested merge to item 18 | 76.7 – merged item | 9c |
| 26 | Create a draft framework/profile for distribution to the community, outline details of how feedback was gathered and used | 53.3 | 31.3 – consider merge with item 27 | 12 |
| 27 | Describe how the framework/profile was validated with the community | 70 | N/A | 12 |
| 28 | Outline the acceptability of the output with the community | 16.7 | 25.1 – consider merge with item 27 | 12 |
| 29 | Describe the lifespan of the framework/profile and outline an update schedule | 60 | 50 | N/A |
| 30 | Describe how to assess outcomes of implementation | 53.3 | 43.8 | N/A |
| 31 | Provide a 'lessons learned' perspective when reporting updates | 46.7 | 26.7 | N/A |
| 32 | Declare the funding source of the framework/profile | 76.7 | N/A | 14 |
| 33 | Declare any conflicts of interest among the developers | 80 | N/A | 15 |
